# Early weight loss predicts poorer prognosis and highlights limitations of predictive energy equations in amyotrophic lateral sclerosis

**DOI:** 10.1007/s00415-026-14093-5

**Published:** 2026-09-08

**Authors:** Amber R. Sewell-Green, Robert Capstick, Cory J. Holdom, Kylie Matthews-Rensch, John Cameron, Pamela A. McCombe, Robert D. Henderson, Shyuan T. Ngo, Frederik J. Steyn

**Affiliations:** 1 University of Queensland Centre for Motor Neuron Disease Research, The University of Queensland, Brisbane, Australia; 2 School of Biomedical Sciences, Faculty of Health, Medicine and Behavioural Sciences, The University of Queensland, Brisbane, Australia; 3 Department of Dietetics and Food Services, Royal Brisbane and Women’s Hospital, Brisbane, Australia; 4 School of Public Health, Faculty of Health, Medicine and Behavioural Sciences, The University of Queensland, Brisbane, Australia; 5 Queensland Centre for Mental Health Research, The Park Centre for Mental Health Treatment, Research and Education, Wacol, Australia; 6 The Prince Charles Hospital, Metro North Hospital and Health Service, Brisbane, Australia; 7 The Frazer Institute, The University of Queensland, Herston, Australia; 8 Department of Neurology, Royal Brisbane and Women’s Hospital, Herston, Australia; 9 Australian Institute for Bioengineering and Nanotechnology, The University of Queensland, Brisbane, Australia

**Keywords:** Amyotrophic lateral sclerosis, Energy balance, Prognosis, Predictive energy equations

## Abstract

**Background:**

Weight loss and malnutrition are associated with poorer outcomes in people living with amyotrophic lateral sclerosis (plwALS). This study investigated the prognostic relevance of early weight loss in plwALS and assessed whether existing energy equations provide useful guidance for nutritional management.

**Methods:**

In this prospective case–control and within-case comparison study (March 2016October 2024), 170 plwALS and 173 non-neurodegenerative disease controls completed baseline anthropometric and metabolic assessment. Participants with ALS were classified according to weight change during the first six months following study inclusion as Weight Gain, Stable Weight, or Weight Loss. Weight-change groups were compared for subsequent changes in anthropometry, functional capacity, and survival. In a nested cohort of 82 plwALS with reported energy intake and repeat assessments, we assessed whether the relationship between reported intake and equation-derived energy requirements was concordant with subsequent weight change.

**Results:**

Early weight loss was associated with continued loss of body weight, fat mass, and fat-free mass, faster functional decline, and increased risk of earlier death relative to Weight Gain (HR=4.78 [95% CI 2.68–8.53], *p*<0.001). Concordance between equation-derived energy requirements, reported energy intake and observed weight trajectory was low (17.3–32.1%). The IBW30(30) equation showed the greatest concordance among participants with Weight Loss, but no equation performed consistently across weight-change groups.

**Conclusions:**

Early weight loss identifies plwALS at increased risk of subsequent weight and body-composition loss, functional decline and earlier death. Existing energy equations proposed for use in ALS show limited concordance with observed weight trajectories, particularly when applied at a single time point. These findings support serial monitoring of nutritional risk and individualized, phenotype-informed dietetic care in ALS.

**Supplementary Information:**

The online version contains supplementary material available at https://doi.org/10.1007/s00415-026-14093-5.

## Introduction

Motor neuron disease (MND) is a progressive neurodegenerative disorder characterized by clinical heterogeneity and multisystem involvement [1, 2]. The most common form of MND, amyotrophic lateral sclerosis (ALS) [2], typically results in death from respiratory failure within 2–5 years following diagnosis [3]. Alongside continued efforts to develop effective disease-modifying therapies, advances in multidisciplinary care have improved clinical support for people living with ALS (plwALS). These advances underscore the importance of evidence-based frameworks that can guide timely, individualized care and improve quality of life [4].

Growing recognition of the non-motor aspects of MND has highlighted metabolism and nutrition as important determinants of disease progression and care [5]. Hypermetabolism, appetite loss, and malnutrition affect up to half of plwALS and are associated with faster functional decline and increased risk of earlier death [5]. Studies consistently report that weight and fat mass loss predict poorer outcomes, whereas higher body mass index (BMI) is associated with slower progression [5]. Such disturbances in energy balance arise through multiple, often co-existing mechanisms, including skeletal muscle and mitochondrial contributions to elevated energy expenditure, alongside reduced intake related to dysphagia, upper-limb weakness and appetite loss [5–7]. Qualitative work has also identified substantial psychological and behavioural barriers to increasing calorie intake and adopting high-calorie diets experienced by plwALS, their carers, and healthcare professionals alike [8, 9]. Recognising these barriers is important, as they may limit the uptake and effectiveness of nutritional interventions. These findings have helped shift clinical attention toward the importance of maintaining body weight and nutritional status throughout the disease course. Despite this progress, further work is needed to determine how best to identify emerging energy imbalance and guide individualized nutritional intervention in clinical practice.

Current nutrition recommendations provide important guidance for dysphagia management, gastrostomy decision-making, and nutritional support in ALS [10]. However, questions surrounding energy requirements and nutritional supplementation remain unresolved. Consequently, substantial variability exists in dietetic practice, with many professionals reporting uncertainty regarding how best to calculate and adjust energy targets for plwALS [11]. A key challenge lies in the heterogeneity of ALS. Variability in metabolic rate, body composition, appetite, functional status, and respiratory involvement complicates standardized approaches to estimating energy requirements. Several ALS-specific equations for predicting energy needs have been developed [12–16], yet their performance across diverse metabolic and clinical profiles remains poorly validated. Refining these tools may strengthen dietetic decision-making and improve the capacity of nutritional support to stabilize weight and respond to changing clinical needs.

Although the prognostic relevance of weight loss in ALS is well established, it remains unclear whether equations proposed for use in ALS generate energy requirement targets that are consistent with subsequent weight trajectory. This exploratory study addressed two linked objectives. First, we examined whether established associations between weight loss and poorer outcomes were reproduced in a longitudinal cohort of plwALS, with particular attention to subsequent changes in fat mass, functional capacity and survival. Second, in a nested cohort with dietary data, we assessed whether the relationship between reported energy intake and requirements derived from equations proposed for use in ALS was concordant with observed six-month weight trajectory. This approach allowed us to characterise the clinical and body-composition outcomes associated with weight loss and evaluate whether existing equations would have provided energy targets consistent with the observed trajectories.

## Methods

### Study design

**Fig. 1:**
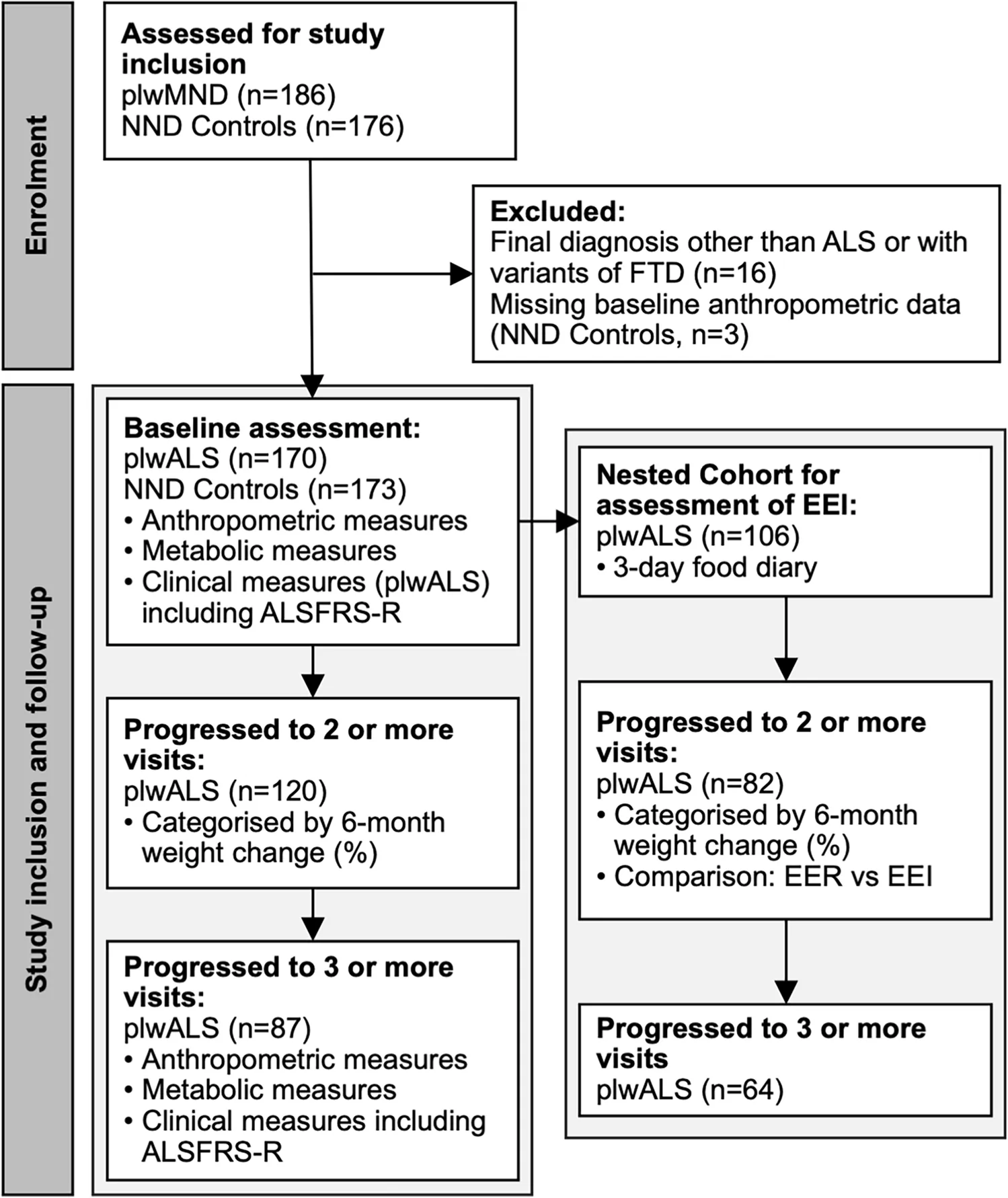
Study enrolment, cohort refinement, and follow-up. Flow diagram showing recruitment of people living with motor neuron disease (plwMND) and non-neurodegenerative disease controls (NND Controls), refinement to the final amyotrophic lateral sclerosis (ALS) cohort, baseline assessment, longitudinal follow-up, and inclusion in the nested cohort used to compare reported estimated energy intake (EEI) with equation-derived estimated energy requirements (EER).*ALSFRS-R* Amyotrophic Lateral Sclerosis Functional Rating Scale-Revised, *EER* estimated energy requirement, *EEI* estimated energy intake, *plwALS* people living with amyotrophic lateral sclerosis

This prospective case–control study was conducted between March 2016 and October 2024. Using convenience sampling, individuals with a diagnosis of MND (*n*=186) were recruited through the Royal Brisbane and Women’s Hospital MND Clinic, and non-neurodegenerative disease controls (*n*=176) were recruited from spouses, relatives and friends of participants with MND (Fig. 1), as described previously [17, 18]. Following baseline assessment, inclusion was restricted to participants meeting the revised Gold Coast diagnostic criteria for ALS [1]. Participants with non-ALS forms of MND or variants of frontotemporal dementia (*n*=16), and controls with missing baseline anthropometric data (*n*=3), were excluded.

At baseline, 170 plwALS and 173 NND Controls completed anthropometric and metabolic assessments. Functional capacity was assessed in plwALS only. Participants were invited to attend follow-up visits for repeated measures every 3–4 months (mean interval, 4.08±2.69 months; range, 2–26 months), with a mean of 4.67±2.78 visits per participant (range, 2–14 visits). A total of 120 plwALS completed two or more visits, representing 1794 participant-months of follow-up.

All participants were asked to complete a 3-day food diary (3DFD) at their initial visit. After quality review by a registered dietitian (ARSG), 106 plwALS had diaries suitable for analysis and were included in a nested cohort. Of these, 82 had repeat visits following baseline assessment, enabling weight categorisation and comparison of measured resting energy expenditure (REE), estimated energy intake (EEI), and weight trajectory as indicators of energy balance.

### Ethical approvals and participant consent

This study was approved by The University of Queensland (2015/HE000022), RBWH (HREC/14/QRBW/495), and Wesley Hospital (1622) human research ethics committees. All participants provided written informed consent prior to participation.

### Anthropometric, metabolic, and clinical assessment

Anthropometric assessment included height, weight, body mass index (BMI), and body composition. Height was measured using a stadiometer at study inclusion. Weight was measured using calibrated scales, while fat mass and fat-free mass were measured using whole-body air displacement plethysmography with the BODPOD Gold Standard system (COSMED, Concord, CA, USA), as previously described [19]. BMI was calculated as weight divided by height in metres squared (kg/m^2^). Measured resting energy expenditure (mREE) was assessed by indirect calorimetry (Quark RMR or Quark Q-NRG; COSMED) under a canopy hood, as described previously [19]. Predicted resting energy expenditure (pREE) was estimated using the “Structure 4” equation reported by Sabounchi et al. [20]. The metabolic index (MI) was calculated as the ratio of mREE to pREE [17].

Clinical assessment included the ALS Functional Rating Scale-Revised (ALSFRS-R) [21], which was used to determine total score and domain subscores: respiratory (questions 10–12), bulbar (questions 1–3), and limb function (questions 4–9). Change in functional capacity (ΔFRS) was calculated as the monthly rate of loss of total ALSFRS-R points since symptom onset [22]. The weight-change category was assigned at the research visit closest to six months after baseline assessment. For the longitudinal and survival analyses, this landmark visit was defined as time zero, and only observations and survival time from this point onward were included.

Longitudinal anthropometric data were used to classify plwALS according to percentage weight change during the first six months after study inclusion. This was guided by the Global Leadership Initiative on Malnutrition (GLIM) weight-loss criteria [23], which define clinically significant weight loss over a six-month period. Given a mean interval of approximately three months between research visits, this window corresponds to approximately two assessments per participant. This framework, validated in ALS cohorts [24], defines weight loss over six months as 0–5% loss, 5–10% loss, and >10% loss. For this study, categories were adapted to reflect observed weight change as: Weight Gain (> 0%), Stable Weight (0–5% loss), and Weight Loss (≥ 5% loss) within the first six months following study inclusion.

### Dietary analysis

Participants completed 3DFDs either as handwritten records using a provided template or digitally via the Research Food Diary app (Xyris Software Australia, Version 6.0.0 202 AF 96 AB 300, 2019) on personal devices. Research staff cross-checked diaries for missing data and nutrition-specific details and reviewed all 3DFDs using predetermined criteria for nutritional accuracy. Each diary captured all food and beverages consumed over three consecutive days, including one weekend day to improve reporting validity [25], with detailed information on food and beverage type, quantity, brand, preparation methods, and timing of intake. Nutritional composition was analysed using FoodWorks10 Professional [26], drawing from AUSFoods, AusBrands, the Australian Food Composition Database, FOODfiles (New Zealand), and the USDA National Nutrient Database for Standard Reference (US) [27]. To account for natural dietary variability, estimated energy intake (EEI) was calculated as average daily energy intake (kcal/day) across the three-day period.

### Predictive energy equation analysis

Estimated energy requirements (EER) were calculated using published equations proposed for use in plwALS [12–16] (Table 1). Equations were selected a priori based on prior application in ALS cohorts, use of routinely available clinical variables, and relevance to clinical practice. Models requiring specialised equipment (e.g., bioelectrical impedance analysis or imaging-based body composition measures), or not previously evaluated in ALS populations, were excluded. For three equations reported by Nakamura et al. [15], BMI thresholds were adapted for the Australian study population by adjusting the ideal body weight (IBW) from 22 kg/m^2^ to 25 kg/m^2^ [28]. We also included an IBW of 30 kg/m^2^ to reflect BMI categories previously associated with prognostic benefit in predominantly Caucasian ALS cohorts [29]. For each equation, estimated energy intake as a percentage of EER (%EEI) was calculated by dividing each participant’s average daily energy intake from the 3DFD by their equation-specific EER. Equation concordance was defined as the proportion of participants whose observed weight-change category (Weight Gain, Stable Weight, or Weight Loss) aligned with their %EEI within a ±10% range, consistent with Australian dietary standards [30]. A %EEI >110% was considered concordant with weight gain, 90–110% with weight maintenance, and <90% with weight loss. Concordance for each equation was expressed as the percentage of participants correctly classified.

**Table 1:** Predictive equations used to calculate daily estimated energy requirements for people living with ALS (plwALS)

Author	Year	Name	EER Equation
Vaisman	2009	Increased REE Equation	507 + 23.65 FFM(kg) + 0.186 EEI(kcal/day)—3.6 × age(yrs)—4.185 × ALSFRS-R (+ 195 if Female)
Ichihara	2012	REE × 1.1	mREE × 1.1
Ichihara	2012	REE × 1.2	mREE × 1.2
Kasarskis	2014	Model 6	HB (kcal) + (55.96 × ALSFRS-6)—168
Kasarskis	2014	Model 8	Owen REE + (BMI × 29.9) + (BMI≤25 × 266) + (55.8 × ALS-6)—1056
Lee	2016	MSJ 4	Mifflin-St Jeor REE x (1.1 + 0.1 × ALSFRS-R Q9)
Lee	2016	MSJ 5	Mifflin-St Jeor REE + 30% of REE if walking or + 20% REE if unable to walk
Shimizu	2017	Shimizu	(1.67 × HB) + (11.8*ALSFRS-R)—680
Nakamura	2023	IBW 22 (25)	25 kcal/kg; Ideal Body Weight = 22 kg/m^2^
Nakamura	2023	IBW 22 (30)	30 kcal/kg; Ideal Body Weight = 22 kg/m^2^
Nakamura	2023	IBW 25 (25)	25 kcal/kg; Ideal Body Weight = Wt @ BMI 25 kg/m^2^ × 25 kcal
Nakamura	2023	IBW 30 (25)	25 kcal/kg; Ideal Body Weight = 30 kg/m^2^
Nakamura	2023	IBW 30 (30)	30 kcal/kg; Ideal Body Weight =30 kg/m^2^

### Statistical methodology

All analyses were performed using RStudio (version 2023.12.1+402). Continuous variables were summarized as mean ± standard deviation (SD), categorical variables as counts and proportions (n, %), and time variables as median and interquartile range (IQR). Case–control and within-case baseline comparisons were assessed using Welch-adjusted Student’s t-tests for continuous variables, Chi-square or Fisher’s exact tests for categorical variables, and Brown-Mood median tests for time variables. Comparisons across three or more groups were assessed using one-way analysis of variance (ANOVA) with post-hoc Tukey’s tests to control for family-wise error across pairwise comparisons. Analyses used the R packages *tidyverse* version 2.0.0 [31], *rstatix* version 0.7.2 [32], and *coin* version 1.4.3 [33]. Additional analyses were stratified by sex to examine potential sex-specific effects.

To assess the effect of weight-change category on subsequent anthropometric measures and ALSFRS-R, separate joint longitudinal-survival models were fitted for weight, BMI, fat-mass, fat-free mass, and ALSFRS-R using the R packages *JM* version 1.5.2 [34], *nlme* version 3.1.168 [35], and *survival* version 3.8.3 [36]. Weight Gain was specified as the reference category. Joint models were used to account for informative dropout associated with death. Each longitudinal submodel included weight-change group, time and the group x time interaction as fixed effects, with participant-level random intercepts and slopes for time. The corresponding survival sub-model used death as the event, specified a Weibull baseline hazard and included sex as a covariate. The longitudinal and survival components were linked through the current modelled value.

Marginal Wald tests were used to evaluate the effect of weight-change category on joint-modelled outcomes, accounting for baseline score. All 120 weight-classified participants were included in each outcome-specific joint model, including participants whose only post-classification observation was the landmark measurement. The landmark measurement and all available subsequent measurements were included in the longitudinal analyses. Model-specific observations varied according to the availability of each outcome. Survival analyses were conducted using Kaplan–Meier estimates with 95% confidence intervals generated using the survival package and visualized using *ggplot2* version 3.5.2 [37], *survminer* version 0.5.0 [38], and *ggpubr* version 0.6.0 [39]. Sex-adjusted Cox proportional hazards regression was used to estimate hazard ratios (HRs) and 95% confidence intervals (CIs) for mortality across weight-change groups, with Weight Gain specified as the reference category. The packages *rvg* version 0.3.5 [40] and *officer* version 0.6.10 [41] were used to export graphs from R. Follow-up for the weight-group joint models and Kaplan–Meier analysis was administratively censored at 36 months after the landmark. Separate secondary joint models were used to estimate associations between the current modelled values of longitudinal anthropometric or functional measures and mortality. Hazard ratios and 95% confidence intervals (CIs) were reported on the original measurement scales and after standardisation. Standardised variables were mean-centred and scaled by one standard deviation to create z-scores (z = [x—μ]/σ), allowing comparison of HRs across measures expressed in different units. Age and sex were included as covariates in the survival sub-model. The Akaike Information Criterion (AIC) and Bayesian Information Criterion (BIC) were used to compare survival sub-models with and without site of disease onset as an additional covariate. The site of disease onset was not retained because its inclusion provided minimal improvement in model fit.

Forest plots depicting concordance of predictive energy equations were generated using *ggplot2* to illustrate the proportion of participants whose weight change category (Weight Gain, Stable Weight, or Weight Loss) was concordant with the relationship between reported EEI and equation-derived EER. Statistical significance was set at α=0.05.

## Results

### Associations between six-month weight change and functional decline

Demographic and baseline clinical characteristics are summarized in Table 2. Compared with NND Controls, plwALS were older (mean, 60.1 years), more frequently male (68.2%), and had comparable baseline weight and body composition. Sex-stratified analyses are presented in Supplementary Table 1. When stratified by sex, fat-free mass was lower in plwALS than NND Controls. Measured resting energy expenditure (mREE) did not differ between groups; however, the metabolic index (MI) was higher in plwALS (p=0.01), an effect observed primarily in males (*p*<0.01). Most plwALS had spinal-onset disease (71.8%), with a median time since symptom onset of 17 months (IQR, 12–24) and a mean ALSFRS-R score of 37.8±5.53 at study inclusion.

**Table 2:** Baseline demographic, metabolic, and clinical characteristics of people living with ALS (plwALS) and non-neurodegenerative disease controls (NND Controls)

	Whole cohort			Nested cohort	
	NND Controls (*n*=173)	plwALS (*n*=170)	*p*	plwALS (*n*=82)	*p*
Demographics					
Age (years)	56.6 (10.7)	60.1 (9.88)	0.002	60.16 (9.50)	0.978
Sex (male, %)	91 (52.6)	116 (68.2)	0.004	58 (70.7)	0.798
Metabolic Measures					
Weight (kg)	82.3 (18.1)	79.5 (17.5)	0.155	80.1 (15.9)	0.813
BMI (kg/m^2^)	27.7 (5.71)	26.6 (5.02)	0.052	26.5 (4.25)	0.872
Fat Mass (kg)	28.8 (12.4)	27.9 (13.1)	0.515	27.6 (11.7)	0.891
Fat Mass (%)	34.2 (10.2)	34.1 (11.7)	0.940	34.0 (10.9)	0.904
Fat Free Mass (kg)	53.2 (11.1)	51.8 (11.4)	0.254	52.4 (11.1)	0.694
Resting Energy Expenditure (kcal/day)^a^	1588 (361)	1630 (402)	0.313	1628 (385)	0.978
Metabolic Index	0.98 (0.13)	1.02 (0.18)	0.010	1.02 (0.18)	0.788
Clinical Measures					
ALSFRS-R Total		37.8 (5.53)		37.8 (5.28)	0.965
Respiratory sub-score		10.8 (1.79)		10.7 (1.84)	0.481
Bulbar sub-score		9.82 (2.43)		9.76 (2.45)	0.846
Lower Limb sub-score		4.84 (2.16)		4.90 (2.06)	0.821
Upper Limb sub-score		6.26 (1.93)		6.40 (1.76)	0.572
∆FRS		0.45 (0.30–0.87)		0.47 (0.33–0.81)	0.689
Time Since Symptom Onset (months)		17 (12.0–24.0)		17 (13.25–23.0)	0.751
Site of Onset (Bulbar, %)		48 (28.2)		24 (29.3)	0.983
Diagnostic Delay (months)		11 (7–18)		11 (7–17)	0.933
Riluzole Usage (%)		97 (57.0)		44 (53.7)	0.708

Data are presented as mean (SD) for continuous variables, n (%) for categorical variables, and median (IQR) for time variables. P values were calculated using Welch-adjusted Student’s t-tests for continuous variables, Chi-square or Fisher’s exact tests for categorical variables, and Brown-Mood median tests for time variables. aBaseline metabolic data were unavailable for n=3 plwALS and n=5 NND Controls in the whole cohort, and n=2 plwALS in the nested cohort

*ALS* amyotrophic lateral sclerosis, *ALSFRS-R* ALS Functional Rating Scale-Revised, *BMI* body mass index, ΔFRS monthly rate of loss of total ALSFRS-R points since symptom onset

**Fig. 2:**
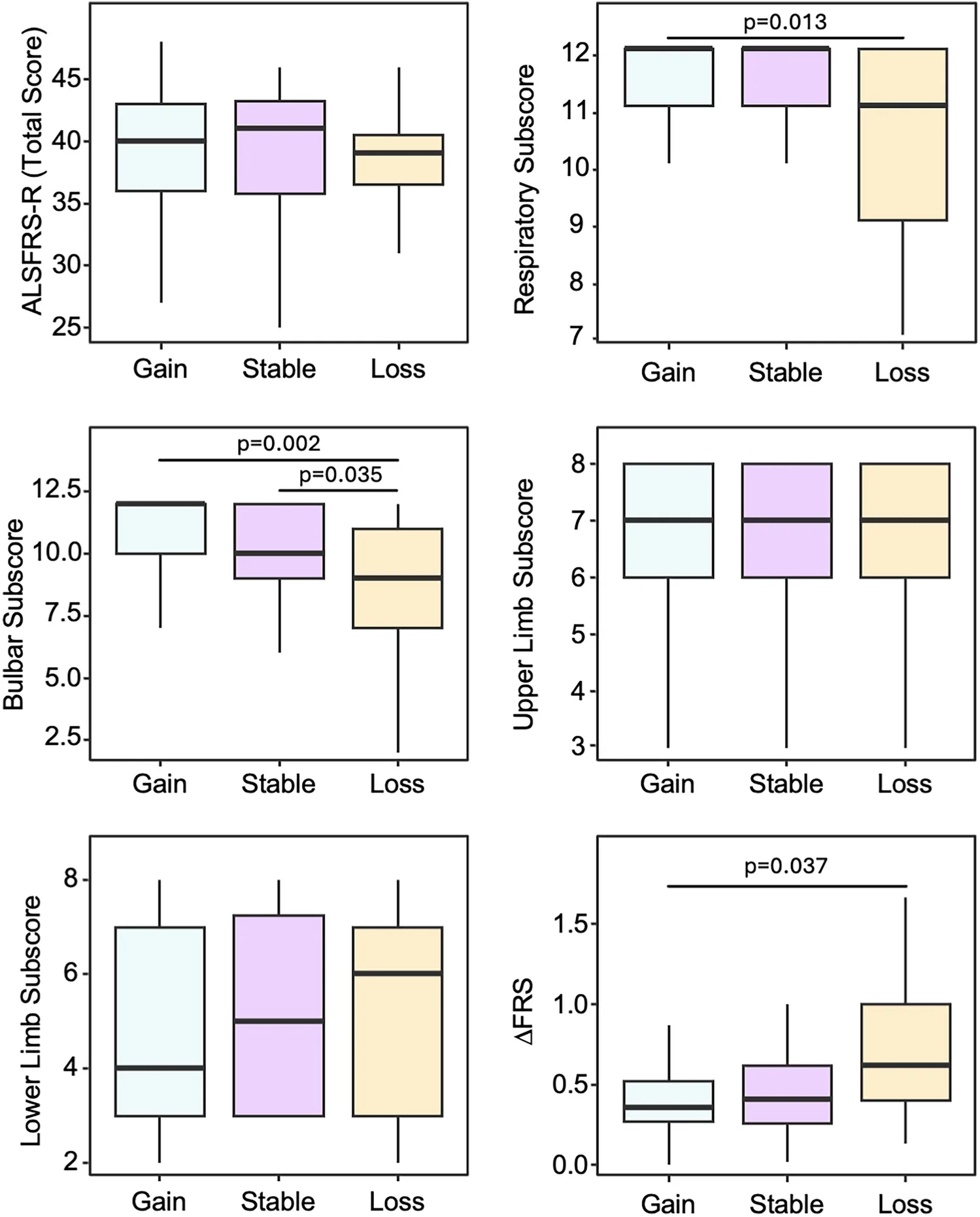
Baseline functional measures by six-month weight-change group. Box-and-whisker plots comparing baseline ALSFRS-R total score, respiratory, bulbar, upper-limb, and lower-limb subscores, and ΔFRS among participants classified as Weight Gain (Gain), Stable Weight (Stable), or Weight Loss (Loss) during the six-month weight-categorization period. Group differences were assessed using one-way ANOVA with post-hoc Tukey’s tests. *ALSFRS-R* Amyotrophic Lateral Sclerosis Functional Rating Scale-Revised, ΔFRS, monthly rate of loss of total ALSFRS-R points since symptom onset

Supplementary Fig. 1 shows weight trajectories during the first six months following baseline assessment for plwALS classified as Weight Gain (*n*=49; mean +2.76 kg [3.53% gain]), Stable Weight (*n*=36; mean − 2.13 kg [2.61% loss]), or Weight Loss (*n*=35; mean − 7.54 kg [10.01% loss]). Demographic and metabolic measures did not differ between Weight Gain, Stable Weight, and Weight Loss groups (Table 3), with sex-stratified comparisons presented in Supplementary Table 2. Baseline anthropometric and clinical measures across weight-change groups are illustrated in Fig. 2. Compared with plwALS who gained weight, those with Weight Loss had lower respiratory (*p*=0.01) and bulbar (*p*<0.01) subscores and a faster rate of functional decline (ΔFRS; 0.62 [IQR, 0.40–1.00] vs 0.36 [IQR, 0.27–0.52] points/month; *p*=0.007).

**Table 3:** Baseline characteristics of people living with ALS (plwALS) by six-month weight-change category, adapted from GLIM weight-loss criteria

	Weight gain (*n*=49)	Stable weight (*n*=36)	Weight loss (*n*=35)	*p*
Demographics				
Age (years)	59.4 (9.81)	59.8 (9.88)	62.6 (9.25)	0.302
Sex (male, %)	37 (75.5)	26 (72.2)	23 (65.7)	0.615
Metabolic measures				
Weight (kg)	79.7 (15.0)	81.6 (15.8)	78.5 (19.7)	0.741
BMI (kg/m^2^)	26.3 (4.03)	26.8 (4.14)	26.2 (5.36)	0.810
Fat Mass (kg)	28.1 (11.0)	27.2 (11.4)	26.0 (12.8)	0.715
Fat Mass (%)	34.5 (9.41)	33.0 (11.5)	32.7 (12.1)	0.708
Fat Free Mass (kg)	51.6 (8.77)	54.3 (12.0)	52.5 (14.0)	0.547
Resting Energy expenditure (kcal/day)^a^	1640 (429)	1634 (389)	1634 (377)	0.997
Metabolic Index	1.03 (0.23)	0.99 (0.15)	1.02 (0.12)	0.627
Clinical measures				
ALSFRS-R Total	39.3 (4.74)	39.0 (5.56)	37.94 (4.83)	0.472
Respiratory sub-score	11.3 (1.18)	10.9 (1.83)	10.27 (1.84)	0.018
Bulbar sub-score	10.6 (2.15)	10.2 (1.90)	8.88 (2.61)	0.003
Lower Limb sub-score	4.67 (2.14)	5.25 (2.05)	5.39 (2.09)	0.253
Upper Limb sub-score	6.51 (1.70)	6.53 (1.87)	6.64 (1.37)	0.941
∆FRS	0.36 (0.27–0.52)	0.42 (0.27–0.65)	0.62 (0.4–1.0)	0.007
Time Since Onset (months)	21 (14–26)	17 (12–24)	14 (11–20.5)	0.018
Site of Onset (Bulbar, %)	10 (20.4)	10 (27.8)	15 (42.9)	0.081
Diagnostic Delay (months)	15 (8.75–18.3)	11 (5.5–16.5)	10 (7–13.5)	0.560
Riluzole Usage (%)	29 (59.2)	19 (52.8)	19 (54.3)	0.822

Participants were categorized by six-month weight change, adapted from GLIM weight-loss criteria, as Weight Gain (> 0%), Stable Weight (0–5% loss), or Weight Loss (≥ 5% loss) during the first six months following study inclusion. Data are presented as mean (SD) for continuous variables, n (%) for categorical variables, and median (IQR) for time variables. P values were calculated using ANOVA with Tukey’s post-hoc tests for continuous variables, Chi-square or Fisher’s exact tests for categorical variables, and Brown-Mood median tests for time variables. ^a^Baseline metabolic data were unavailable for n=2 participants with ALS

*ALS* amyotrophic lateral sclerosis, *ALSFRS-R* ALS Functional Rating Scale-Revised, *BMI* body mass index, ΔFRS, monthly rate of loss of total ALSFRS-R points since symptom onset

### Weight-change trajectory and longitudinal changes in body composition, functional decline and survival

**Fig. 3:**
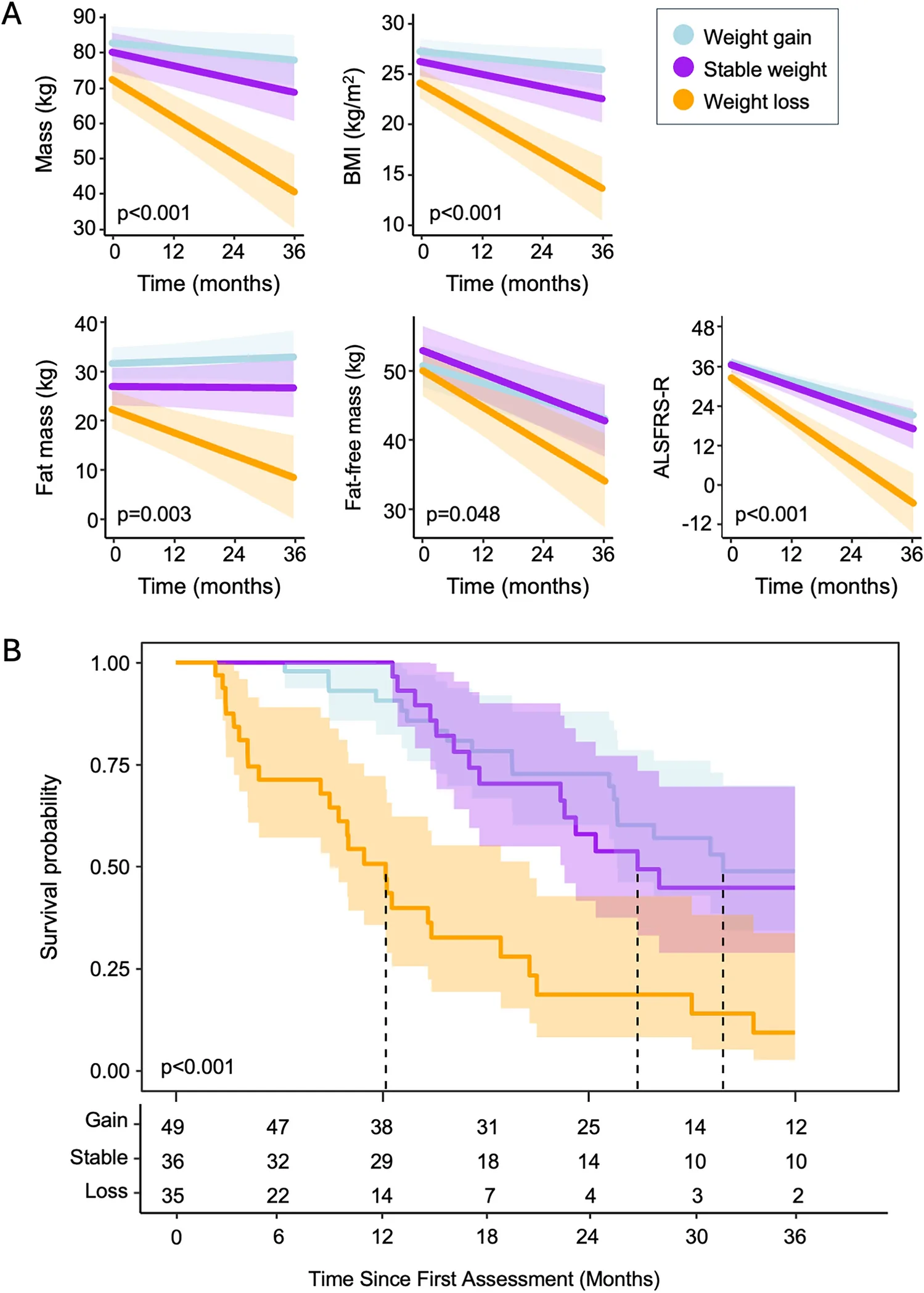
Longitudinal trajectories and survival by six-month weight-change group. Time zero represents the visit closest to six months after baseline assessment, at which participants were classified as Weight Gain, Stable Weight or Weight Loss based on weight change during the preceding six-month monitoring period. **A** Modelled trajectories of body weight, body mass index (BMI), fat mass, fat-free mass and ALSFRS-R score from time zero. Lines show modelled means and shaded areas show 95% confidence intervals derived from linear mixed-effects joint models. **B** Kaplan–Meier survival curves from time zero, administratively censored at 36 months, with numbers at risk shown at six-month intervals. *ALSFRS-R* Amyotrophic Lateral Sclerosis Functional Rating Scale-Revised, *BMI* body mass index

Changes in weight, body composition, and function after the six-month weight-categorization period were assessed using linear mixed-effects joint models (Fig. 3A, Table 4). Compared with participants who maintained Stable Weight or experienced Weight Gain, those with Weight Loss showed greater continued declines in body weight and BMI. Participants with Weight Loss also showed ongoing declines in fat mass, and fat-free-mass trajectories differed between weight-change groups (*p*=0.048), with the greatest modelled decline observed in the Weight Loss group. Functional decline was also greater in the Weight Loss group, reflected by a steeper reduction in ALSFRS-R scores over follow-up (*p*<0.001).

**Table 4:** Longitudinal slopes for anthropometric and clinical measures by six-month weight-change category, derived from linear mixed-effects joint models

	Slopes (95% CI)			
	Weight Gain (*n*=49)	Stable Weight (*n*=36)	Weight Loss (*n*=35)	*p*
Weight (kg)	−0.133 (− 0.267 to 0.001)	−0.311 (− 0.644 to 0.023)	−0.873 (− 1.275 to − 0.471)	<0.001
BMI (kg/m^2^)	−0.049 (− 0.093 to − 0.004)	−0.102 (− 0.213 to 0.008)	−0.290 (− 0.422 to − 0.158)	<0.001
Fat Mass (kg)	0.037 (− 0.083 to 0.156)	−0.007 (− 0.304 to 0.289)	−0.386 (− 0.752 to − 0.019)	0.003
Fat-Free Mass (kg)	−0.214 (− 0.300 to − 0.127)	−0.279 (− 0.492 to − 0.066)	−0.442 (− 0.703 to − 0.181)	0.048
ALSFRS-R	−0.434 (− 0.560 to − 0.308)	−0.533 (− 0.862 to − 0.203)	−1.052 (− 1.451 to − 0.654)	<0.001

Model-derived slopes represent longitudinal change in anthropometric and clinical measures after the six-month weight-categorization period. Slopes were estimated using linear mixed-effects joint models with time as a fixed effect and participant-level random intercepts and slopes. *ALSFRS-R* Amyotrophic Lateral Sclerosis Functional Rating Scale-Revised, *BMI* body mass index

During the 36-month follow-up period, participants with Weight Loss had shorter median survival (12.2 months [IQR, 8.94–20.6]) than those classified as Stable Weight (26.8 months [IQR, 22.6–36.0]) or Weight Gain (31.8 months [IQR, 25.7–36.0]), corresponding to an approximately five-fold higher mortality hazard of earlier death when compared to the Weight Gain reference group (HR=4.78 [95% CI 2.68–8.53], *p*<0.001; Fig. 3B). Further survival analyses were conducted using joint models adjusted for age and sex (Table 5). Hazard ratios were standardized to allow direct comparison across variables, with ALSFRS-R included as a reference prognostic marker. Across anthropometric measures, higher longitudinal values were associated with lower mortality risk. Higher body weight showed the strongest anthropometric association with lower mortality risk (standardized HR=0.42 [95% CI 0.28–0.63]), while higher fat mass was associated with a modest, but significant reduction in risk (standardized HR=0.63 [95% CI 0.47–0.85]).

**Table 5:** Associations between longitudinal anthropometric and functional measures and mortality in joint models

	Absolute	Standardised	
	HR (95% CI)	HR (95% CI)	*p*
Weight (kg)	0.96 [0.94 to 0.98]	0.42 [0.28 to 0.63]	<0.001
BMI (kg/m^2^)	0.86 [0.80 to 0.92]	0.45 [0.31 to 0.65]	<0.001
Fat Mass (kg)	0.96 [0.94 to 0.99]	0.63 [0.47 to 0.86]	0.033
Fat-Free Mass (kg)	0.95 [0.92 to 0.98]	0.48 [0.32 to 0.71]	<0.001
ALSFRS-R	0.89 [0.86 to 0.93]	0.47 [0.36 to 0.61]	0.016

Hazard ratios were estimated from joint models with age and sex included as covariates in the survival component and time included as a participant-level random effect in the linear mixed-effects component. Data are presented as hazard ratio [95% confidence interval] for absolute values and standardized values. Standardized estimates represent the change in mortality risk per 1-SD increase in each variable. Statistical significance was set at *p*<0.05. *ALSFRS-R* Amyotrophic Lateral Sclerosis Functional Rating Scale-Revised, *BMI* body mass index

### Concordance of energy-prediction equations with observed weight trajectories

Baseline characteristics of the nested cohort used for the predictive-equation analysis are summarized in Table 6. No significant differences in demographic, baseline anthropometric, or metabolic measures were observed between weight-change groups. Although plwALS with Weight Loss had greater bulbar and respiratory involvement, estimated energy intake at study inclusion was similar across groups (mean range, 2314–2403 kcal/day; *p*=0.84), and remained comparable when stratified by sex (Supplementary Table 3).

**Table 6:** Baseline characteristics of people living with ALS (plwALS) in the nested cohort by six-month weight-change category

	Weight Gain (*n*=31)	Stable Weight (*n*=31)	Weight Loss (*n*=20)		*p*
Demographics					
Sex (male, %)	23 (74.2)	23 (74.2)	12 (60.0)	0.479	
Age (years)	57.4 (8.40)	61.7 (9.89)	62.2 (9.91)	0.111	
Metabolic Factors					
Weight (kg)	81.2 (13.4)	79.2 (16.0)	79.7 (19.7)	0.873	
BMI (kg/m^2^)	26.8 (3.59)	26.17 (4.35)	26.6 (5.15)	0.868	
Fat Mass (kg)	29.6 (10.2)	25.8 (12.1)	27.4 (13.4)	0.448	
Fat Mass (%)	35.9 (9.16)	31.9 (11.8)	34.1 (12.1)	0.353	
Fat Free Mass (kg)	51.7 (9.01)	53.34 (11.2)	52.2 (14.0)	0.834	
Estimated Energy Intake (kcal/day)	2314 (579)	2403 (655)	2381 (601)	0.844	
Resting Energy Expenditure (kcal/day)^a^	1692 (372)	1594 (395)	1584 (395)	0.525	
Metabolic index	1.07 (0.22)	0.98 (0.15)	0.99 (0.13)	0.163	
Clinical Factors					
ALSFRS-R	38.1 (5.55)	38.0 (5.06)	37.0 (5.36)	0.750	
Respiratory sub-score	11.1 (1.54)	10.8 (1.67)	9.75 (2.24)	0.028	
Bulbar sub-score	10.7 (2.01)	9.52 (2.13)	8.75 (3.11)	0.019	
Lower Limb sub-score	4.10 (1.87)	5.23 (2.14)	5.65 (1.90)	0.016	
Upper Limb sub-score	6.32 (2.06)	6.42 (1.65)	6.5 (1.50)	0.940	
∆FRS	0.36 (0.25–0.59)	0.50 (0.33–0.74)	0.75 (0.43–1.26)	0.056	
Time Since Onset (months)	21.0 (15.5–25.3)	17 (12.5–21.5)	14.5 (11.0–17.5)	0.003	
Site of Onset (Bulbar, %)	5 (16.1)	12 (38.7)	7 (35.0)	0.120	
Diagnostic Delay (months)	16 (9.00–17.1)	11.0 (5.50–15.5)	9.5 (6.0–12.3)	0.513	
Riluzole Usage (%)	19 (61.3)	16 (51.6)	9 (45.0)	0.501	

Participants in the nested cohort were categorized by six-month weight change, adapted from GLIM weight-loss criteria, as Weight Gain (> 0%), Stable Weight (0–5% loss), or Weight Loss (≥ 5% loss) during the first six months following study inclusion. Data are presented as mean (SD) for continuous variables, n (%) for categorical variables, and median (IQR) for time variables. P values were calculated using ANOVA with Tukey’s post-hoc tests for continuous variables, Chi-square or Fisher’s exact tests for categorical variables, and Brown-Mood median tests for time variables. ^a^Baseline metabolic data were unavailable for *n*=2 participants with ALS. *ALS* amyotrophic lateral sclerosis, *ALSFRS-R* ALS Functional Rating Scale-Revised, *BMI* body mass index, ΔFRS, monthly rate of loss of total ALSFRS-R points since symptom onset

**Fig. 4:**
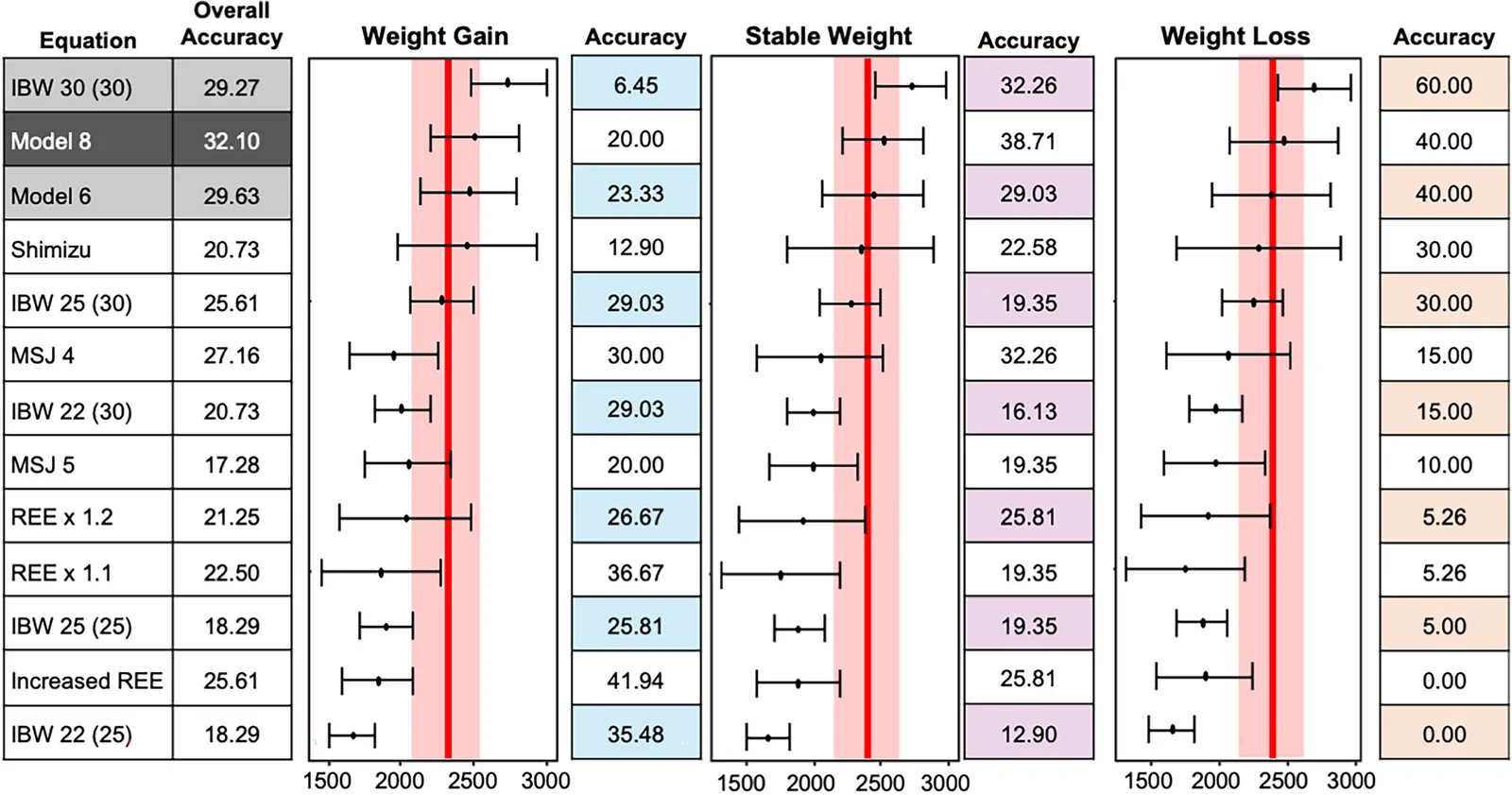
Reported energy intake and equation-derived energy requirements across weight-change groups. Thirteen equations were evaluated in the nested cohort to determine whether equation-derived estimated energy requirements (EER) aligned with reported estimated energy intake (EEI) and observed six-month weight-change category. For each equation, predicted EER is shown relative to EEI derived from 3-day food diaries (red shaded area). Concordance was defined as alignment between the relationship of equation-derived EER to reported EEI and the observed weight-change category, using a ±10% threshold for expected weight maintenance. *EEI* estimated energy intake, *EER* estimated energy requirement, *IBW* ideal body weight, *MSJ* Mifflin-St Jeor, *REE* resting energy expenditure

Equation performance was assessed by determining whether each equation-derived EER was concordant with reported energy intake and the observed six-month weight-change category, with raw values presented in Supplementary Table 4. Overall concordance with the observed weight-change category was low (Fig. 4), ranging from 17.3% for the MSJ5 Eq. (14) to 32.1% for Model 8 [13, 14]. The adapted IBW30(30) ratio [15] showed the greatest concordance among participants with Weight Loss (60.0%), although it tended to generate higher energy targets for participants maintaining Stable Weight or experiencing Weight Gain. Conversely, the Increased REE [13, 16] generated estimated requirements below reported intake across all weight-change groups. While this was concordant with observed Weight Gain, its application in participants with Weight Loss would have suggested adequate or excess reported intake, despite eventual weight loss.

## Discussion

In this prospective case–control and within-case study of plwALS, we examined six-month weight change and assessed whether the relationship between reported energy intake and equation-derived energy requirements was concordant with observed weight trajectories. Six-month weight loss identified a clinically vulnerable subgroup characterised by ongoing loss of body weight and fat mass, faster functional decline and reduced survival. The parallel decline in body weight and fat mass is consistent with sustained negative energy balance, rather than weight loss attributable solely to loss of muscle mass and supports previous evidence linking nutritional status with prognosis in ALS [5, 42]. Importantly, these differences emerged despite comparable baseline body weight, BMI, fat mass, fat-free mass and measured resting energy expenditure across weight-change groups. These findings reinforce the clinical importance of monitoring weight trajectory in plwALS to identify individuals who may benefit from timely dietetic assessment. Against this clinical context, the limited concordance between reported energy intake, equation-derived requirements and subsequent weight trajectory has important implications for nutritional management. Identifying nutritional risk is necessary, but currently available equations provide limited guidance for translating that risk into individualised energy targets.

The limited concordance of current predictive equations may reflect the marked metabolic and phenotypic heterogeneity of ALS [3]. In our cohort, six-month weight loss was associated with greater bulbar and respiratory involvement and faster functional decline, suggesting that energy requirements are unlikely to be adequately represented by a single equation based primarily on static anthropometric or functional variables [43]. Non-resting components of total daily energy expenditure may also contribute substantially to individual energy needs [44]. These components are likely to vary according to physical activity, movement efficiency, respiratory muscle work and disease stage, but remain incompletely characterised in ALS [45]. Respiratory involvement may be particularly relevant, with prior studies suggesting that impaired respiratory function can increase metabolic demand and contribute to underestimation of energy requirements by standard formulae [46, 47]. Predictive equations remain clinically useful when indirect calorimetry or doubly labelled water are unavailable [43]; however, our findings indicate that they should be interpreted alongside weight trajectory, body composition, respiratory status and functional decline. We note, also, that the more modest difference in metabolic index between plwALS and controls may partly reflect the selection of Structure 4 as the reference equation, as metabolic index estimates vary with the equation used [17].

### Clinical implications

These findings support a more individualized approach to nutritional assessment in ALS. Computational modelling indicates that site of onset, age, respiratory function, and rate of ALSFRS-R decline predispose plwALS to severe weight loss [48]. These factors align with our findings, where individuals with early weight loss had greater respiratory and bulbar involvement and faster functional decline. Stratifying plwALS according to clinical and metabolic profiles may, therefore, help identify individuals who require more intensive nutritional support, closer dietetic monitoring, or higher energy targets to stabilize weight. Within our study, sex-adjusted equations, such as Harris-Benedict and Mifflin-St Jeor [13], showed greater concordance in participants with 0–5% weight loss and have previously been reported to align more closely with energy-expenditure estimates obtained using indirect calorimetry or doubly labelled water [49]. In contrast, simple ratio-based equations, including the IBW30 approach adapted from Nakamura et al. [15], appeared more concordant in participants with weight loss, but generated higher energy targets for participants who were weight-stable or gained weight. These findings argue against universal application of any single equation and support staged, phenotype-informed dietetic assessment. This is particularly important given evidence that body weight and BMI may have non-linear relationships with prognosis in ALS [29], such that the clinical goal should be prevention of harmful weight and fat mass loss rather than indiscriminate weight gain. In practice, implementing this phenotype-informed approach may be constrained by variation in dietetic practice, geographical access to specialist nutritional services and limited ALS-specific guidance for setting and adjusting energy targets [11, 50–52].

Beyond total energy prescription, macronutrient composition and substrate utilization may also influence weight trajectories and clinical outcomes in ALS. Addressing negative energy balance and preserving fat mass are plausible targets for nutritional intervention [53]. Randomized controlled trials have shown that high-energy diets can stabilize weight and improve outcomes in some plwALS, particularly those with faster-progressing disease [54]; however, other calorie-based interventions have reported inconsistent effects [11, 55]. This may reflect differences in disease stage, nutritional risk, metabolic phenotype, or the substrates contributing energy. Evidence of impaired metabolic flexibility, glucose intolerance, and altered lactate metabolism in ALS supports the possibility that disrupted substrate utilization contributes to energy imbalance [5, 6]. Observational studies linking higher fat intake with longer survival [56], together with epidemiological evidence associating pre-existing diabetes or insulin resistance with reduced ALS risk [57], further suggest that lipid-derived substrates may be relevant to metabolic adaptation in ALS. However, current evidence remains insufficient to define optimal macronutrient targets, and guidelines do not identify specific macronutrient strategies to prevent or reverse weight loss in plwALS [11, 58]. Ongoing trials, including the Sheffield-led OptiCALS randomised controlled trial of an individualised high-calorie dietary intervention, are designed to determine whether modifying nutritional status influences functional and nutritional outcomes in ALS [59]. Future intervention studies should, therefore, consider both total energy prescription and whether dietary composition can be tailored to metabolic phenotype, weight trajectory, and disease stage.

Improved ALS-specific approaches to estimating energy requirements are needed. Rather than relying on universal equations, future models should account for disease-related heterogeneity, including hypermetabolism, respiratory involvement, altered movement efficiency, and dynamic changes in body composition [42]. In other metabolically heterogeneous populations, incorporating activity, stress, or injury factors alongside resting energy expenditure has improved estimation of energy requirements [60, 61]. Similar approaches may be relevant in ALS, provided that adjustment factors are derived from disease-specific data and validated against appropriate reference measures. Methods, such as accelerometry, whole-room calorimetry, and doubly labelled water, may help quantify free-living energy expenditure and validate scalable prediction models [62, 63]. Integrating these approaches with longitudinal measures of weight, body composition, respiratory function, and functional decline may improve identification of patients at risk of ongoing weight and fat mass loss before clinically significant malnutrition develops.

### Limitations

This study has several limitations. First, the observational design precludes causal inference regarding the relationships between weight trajectory, metabolic status, dietary intake and survival. Weight loss may, therefore, reflect, rather than drive, a more aggressive disease course. Determining whether modifying weight trajectory improves clinical outcomes will require evidence from randomised controlled trials. Dietary intake was assessed using a three-day food diary at study inclusion and was not repeated during the six-month weight-classification period. The equation analysis, therefore, could not account for subsequent changes in intake, physical activity or other components of total energy expenditure. Food diaries are also subject to reporting errors. Accordingly, the observed concordance reflects the relationship between baseline reported intake, equation-derived requirements and subsequent weight trajectory; it does not constitute formal validation against measured total energy expenditure. The cohort demonstrated greater weight stability than some national and international ALS populations, with 13% meeting GLIM criteria for severe malnutrition compared with 38–53% reported elsewhere [24, 64]. This may limit generalisability to more advanced or nutritionally compromised populations. In addition, weight-change classification required participants to survive and remain under observation through the six-month landmark; the landmark analyses are, therefore, conditional on reaching this time point and may be subject to selection bias. Finally, several equations assessed in this study have not undergone formal validation against measured total energy expenditure in ALS or in Australian ALS cohorts.

## Conclusions

Taken together, these findings demonstrate that six-month weight loss identifies a clinically vulnerable ALS subgroup characterised by ongoing loss of body weight and fat mass, faster functional decline and poorer survival. Although weight and fat-mass loss are consistent with sustained negative energy balance, current predictive energy equations showed limited concordance with reported energy intake and observed weight trajectories. Equation-derived estimates, particularly when applied at a single time point, should, therefore, not be interpreted in isolation when guiding individualised nutritional management in ALS. These results support earlier identification of nutritional risk, serial monitoring of weight trajectory and body composition, and more phenotype-informed dietetic care. Future studies should determine whether ALS-specific models incorporating weight trajectory, body composition, respiratory function, functional decline and measures of free-living energy expenditure can improve nutritional management in ALS.

## Supplementary Information

Below is the link to the electronic supplementary material.

Supplementary file1 (DOCX 111 KB)

## Data Availability

All data generated or analysed during this study are included in this published article and its supplementary information files. Additional data or clarification can be made available from the corresponding author upon reasonable request.
