# Supplementary material for: Early weight loss predicts poorer prognosis and highlights limitations of predictive energy equations in amyotrophic lateral sclerosis
Source: J Neurol. 2026 Sep 8;273(10):575. doi: 10.1007/s00415-026-14093-5 (PMC13553676; doi:10.1007/s00415-026-14093-5)
Supplement: Supplementary file 1 [file 415_2026_14093_MOESM1_ESM.docx]

**Supplementary Figures**

**Supplementary Figure 1. Modelled trajectories of weight, body composition, and function including the six-month weight-categorization period.** Modelled trajectories of weight, body mass index (BMI), fat mass, fat-free mass, and ALSFRS-R scores including the first six months of follow-up used to classify participants as Weight Gain, Stable Weight, or Weight Loss. Abbreviations: ALSFRS-R, Amyotrophic Lateral Sclerosis Functional Rating Scale-Revised; BMI, body mass index.

**Supplementary Tables**

| **Supplementary Table 1.** Baseline demographic, metabolic, and clinical characteristics of people living with ALS (plwALS) and non-neurodegenerative disease controls (NND Controls), stratified by sex. | | | | | | | | | | | | | | |  |  |
| --- | --- | --- | --- | --- | --- | --- | --- | --- | --- | --- | --- | --- | --- | --- | --- | --- |
|  | **Whole Cohort** | | | | | | |  | **Nested Cohort** | | | | | | |  |
|  | **Male** | | |  | **Female** | | | |  | **Male** | |  | **Female** | | | |
|  | **NND Controls (n=89)** | **plwALS (n=127)** | **p** |  | **NND Controls (n=79)** | **plwALS (n=53)** | **p** | |  | **plwALS (n=58)** | **p** |  | **plwALS (n=24)** | **p** | | |
| **Demographics** |  |  |  |  |  |  |  | |  |  |  |  |  |  | | |
| Age (years) | 57.8 (11.1) | 59.4 (9.90) | 0.287 |  | 55.2 (10.1) | 61.7 (9.76) | <0.001 | |  | 59.7 (10.1) | 0.827 |  | 61.2 (8.10) | 0.801 | | |
| **Metabolic Factors** |  |  |  |  |  |  |  | |  |  |  |  |  |  | | |
| Weight (kg) | 88.9 (14.51) | 83.3 (16.2) | 0.010 |  | 75.0 (19.0) | 71.5 (17.7) | 0.274 | |  | 83.0 (16.2) | 0.903 |  | 73.0 (13.1) | 0.669 | | |
| BMI (kg/m^2^) | 27.9 (4.19) | 26.4 (4.49) | 0.014 |  | 27.5 (7.04) | 26.9 (6.03) | 0.631 | |  | 26.4 (4.39) | 0.939 |  | 26.8 (3.95) | 0.892 | | |
| Fat Mass (kg) | 27.1 (10.8) | 26.0 (12.4) | 0.468 |  | 30.6 (13.9) | 32.0 (13.8) | 0.568 | |  | 25.9 (12.3) | 0.990 |  | 31.8 (9.12) | 0.941 | | |
| Fat Mass (%) | 29.7 (7.66) | 29.9 (10.2) | 0.850 |  | 39.3 (10.5) | 43.2 (9.40) | 0.024 | |  | 30.2 (10.2) | 0.851 |  | 43.0 (6.76) | 0.903 | | |
| Fat Free Mass (kg) | 61.8 (7.21) | 57.4 (8.21) | <0.001 |  | 43.7 (5.46) | 40.0 (7.40) | 0.002 | |  | 57.1 (9.04) | 0.839 |  | 41.2 (6.65) | 0.469 | | |
| Resting Energy Expenditure (kcal/day) | 1814 (274) | 1795 (318) | 0.652 |  | 1344 (277) | 1261 (321) | 0.126 | |  | 1760 (348) | 0.519 |  | 1281 (300) | 0.758 | | |
| Metabolic Index (S4) | 1.01 (0.10) | 1.06 (0.16) | 0.006 |  | 0.94 (0.15) | 0.94 (0.19) | 0.848 | |  | 1.04 (0.19) | 0.581 |  | 0.94 (0.14) | 0.950 | | |
| **Clinical Factors** |  |  |  |  |  |  |  | |  |  |  |  |  |  | | |
| ALSFRS-R |  | 38.3 (5.47) |  |  |  | 36.8 (5.58) |  | |  | 38.4 (4.81) | 0.873 |  | 36.3 (6.11) | 0.710 | | |
| Respiratory sub-score |  | 10.8 (1.79) |  |  |  | 10.9 (1.81) |  | |  | 10.8 (1.67) | 0.942 |  | 10.4 (2.22) | 0.313 | | |
| Bulbar sub-score |  | 9.75 (2.47) |  |  |  | 9.96 (2.34) |  | |  | 9.66 (2.69) | 0.819 |  | 10.0 (1.79) | 0.939 | | |
| Lower Limb sub-score |  | 5.14 (2.06) |  |  |  | 4.20 (2.24) |  | |  | 5.10 (2.02) | 0.908 |  | 4.42 (2.12) | 0.690 | | |
| Upper Limb sub-score |  | 6.35 (2.02) |  |  |  | 6.09 (1.73) |  | |  | 6.55 (1.71) | 0.484 |  | 6.04 (1.88) | 0.910 | | |
| ∆FRS |  | 0.43 (0.30-0.81) |  |  |  | 0.53 (0.32-0.93) |  | |  | 0.43 (0.30-0.72) | 0.957 |  | 0.69 (0.42-1.09) | 0.323 | | |
| Time Since Symptom Onset (months) |  | 17 (11.5-25) |  |  |  | 19 (15-23) |  | |  | 17 (12.3-24.0) | 0.870 |  | 17 (14-21) | 0.244 | | |
| Diagnostic Delay (months) |  | 10 (7-18) |  |  |  | 12 (8-17) |  | |  | 11 (6-18) | 0.746 |  | 12 (8.5-14.3) | 0.288 | | |
| Riluzole Usage (%) |  | 67 (57.8) |  |  |  | 30 (55.6) |  | |  | 34 (58.6) | 1.000 |  | 10 (41.7) | 0.375 | | |
| Bulbar Onset (%) |  | 33 (28.5) |  |  |  | 15 (27.8) |  | |  | 17 (29.3) | 1.000 |  | 7 (29.2) | 1.000 | | |
| Data are presented as mean (SD) for continuous variables, n (%) for categorical variables, and median (IQR) for time variables. P values were calculated using Welch-adjusted Student’s t-tests for continuous variables, Chi-square or Fisher’s exact tests for categorical variables, and Brown-Mood median tests for time variables. ^a^Baseline metabolic data were unavailable for n=3 plwALS and n=5 NND Controls in the whole cohort, and n=2 plwALS in the nested cohort. Abbreviations: ALS, amyotrophic lateral sclerosis; ALSFRS-R, ALS Functional Rating Scale-Revised; BMI, body mass index; ΔFRS, monthly rate of loss of total ALSFRS-R points since symptom onset. | | | | | | | | | | | | | | |  |  |

| **Supplementary Table 2.** Baseline characteristics of people living with ALS (plwALS) by six-month weight-change category adapted from GLIM weight-loss criteria, stratified by sex. | | | | | | | | | | |
| --- | --- | --- | --- | --- | --- | --- | --- | --- | --- | --- |
|  |  | **Male** | | | |  | **Female** | | | |
|  |  | **Weight gain (n=37)** | **Stable weight**  **(n=26)** | **Weight loss (n=23)** | **p** |  | **Weight gain (n=12)** | **Stable weight (n=10)** | **Weight loss (n=12)** | **p** |
| **Demographics** |  |  |  |  |  |  |  |  |  |  |
| Age (years) |  | 59.4 (10.4) | 59.3 (11.0) | 60.3 (8.64) | 0.930 |  | 59.5 (8.20) | 61.2 (6.49) | 67.0 (9.13) | 0.076 |
| **Metabolic Factors** |  |  |  |  |  |  |  |  |  |  |
| Weight (kg) |  | 83.2 (14.8) | 85.5 (16.4) | 83.3 (19.3) | 0.843 |  | 68.9 (10.2) | 71.4 (8.28) | 69.4 (17.6) | 0.894 |
| BMI (kg/m^2^) |  | 26.5 (4.36) | 27.0 (4.37) | 26.2 (5.47) | 0.818 |  | 25.5 (2.81) | 26.3 (3.63) | 26.3 (5.36) | 0.873 |
| Fat Mass (kg) |  | 28.3 (11.7) | 25.6 (12.4) | 23.4 (13.3) | 0.322 |  | 27.6 (9.24) | 31.4 (6.77) | 31.0 (10.5) | 0.548 |
| Fat Mass (%) |  | 33.0 (9.38) | 28.8 (10.3) | 26.7 (9.92) | 0.048 |  | 39.2 (8.16) | 43.8 (6.43) | 44.1 (6.30) | 0.193 |
| Fat Free Mass (kg) |  | 54.9 (7.17) | 59.9 (8.80) | 59.9 (9.85) | 0.031 |  | 41.3 (3.64) | 39.9 (5.30) | 38.4 (8.88) | 0.557 |
| Resting Metabolic Rate (kcal/day) |  | 1786 (322) | 1796 (314) | 1818 (294) | 0.929 |  | 1148 (381) | 1213 (209) | 1249 (194) | 0.691 |
| Metabolic Index (S4) |  | 1.08 (0.20) | 1.03 (0.14) | 1.05 (0.11) | 0.387 |  | 0.85 (0.25) | 0.90 (0.13) | 0.97 (0.13) | 0.274 |
| **Clinical Factors** |  |  |  |  |  |  |  |  |  |  |
| ALSFRS-R |  | 39.4 (4.26) | 39.4 (6.03) | 39.1 (3.88) | 0.968 |  | 38.8 (6.18) | 38.0 (4.16) | 35.7 (5.79) | 0.377 |
| Respiratory sub-score |  | 11.2 (1.28) | 10.9 (1.95) | 10.4 (1.57) | 0.242 |  | 11.8 (0.62) | 11.0 (1.56) | 10.0 (2.30) | 0.046 |
| Bulbar sub-score |  | 10.7 (1.96) | 10.0 (2.10) | 9.14 (2.57) | 0.044 |  | 10.5 (2.75) | 10.8 (1.14) | 8.42 (2.71) | 0.045 |
| Lower Limb sub-score |  | 4.84 (2.13) | 5.65 (2.02) | 5.71 (1.76) | 0.168 |  | 4.17 (2.21) | 4.20 (1.81) | 4.83 (2.55) | 0.721 |
| Upper Limb sub-score |  | 6.43 (1.63) | 6.58 (1.88) | 6.91 (1.37) | 0.579 |  | 6.75 (1.96) | 6.40 (1.96) | 6.17 (1.27) | 0.715 |
| Bulbar Onset (%) |  | 7 (18.9) | 8 (30.8) | 10 (43.5) | 0.122 |  | 3 (25.0) | 2 (20.0) | 5 (41.7) | 0.639 |
| ∆FRS |  | 0.33 (0.27-5.0) | 0.40 (0.22-0.56) | 0.62 (0.39-1.06) | 0.035 |  | 0.44 (0.28-0.90) | 0.53 (0.32-0.72) | 0.60 (0.45-0.92) | 0.724 |
| Time Since Symptom Onset (months) |  | 21 (14.0-28.0) | 16.0 (12.0-24.0) | 13.0 (8.5-20.0) | 0.029 |  | 19.5 (16.3-21.3) | 17.5 (15.5-23.5) | 16.5 (14.0-23.0) | 0.630 |
| Diagnostic Delay (months) |  | 15.5 (9.0-21.5) | 11.0 (5.0-17.0) | 8.0 (6.5-11.5) | 0.031 |  | 11 (7.75-17.0) | 12 (7.25-15.3) | 12 (11.5-16.3) | 0.996 |
| Riluzole Usage (%) |  | 23 (62.2) | 14 (53.9) | 13 (56.5) | 0.792 |  | 6 (50.0) | 5 (50.0) | 6 (50.0) | 1.000 |
| Participants were categorized by six-month weight change, adapted from GLIM weight-loss criteria, as Weight Gain (>0%), Stable Weight (0-5% loss), or Weight Loss (≥5% loss) during the first six months following study inclusion. Data are presented as mean (SD) for continuous variables, n (%) for categorical variables, and median (IQR) for time variables. P values were calculated using ANOVA with Tukey’s post-hoc tests for continuous variables, Chi-square or Fisher’s exact tests for categorical variables, and Brown-Mood median tests for time variables. ^a^Baseline metabolic data were unavailable for n=2 participants with ALS. Abbreviations: ALS, amyotrophic lateral sclerosis; ALSFRS-R, ALS Functional Rating Scale-Revised; BMI, body mass index; ΔFRS, monthly rate of loss of total ALSFRS-R points since symptom onset. | | | | | | | | | | |

| **Supplementary Table 3.** Baseline characteristics of people living with ALS (plwALS) in the nested cohort by six-month weight-change category, stratified by sex. | | | | | | | | | |
| --- | --- | --- | --- | --- | --- | --- | --- | --- | --- |
|  | **Male** | | | |  | **Female** | | | |
| **Demographics** | **Weight gain (n=23)** | **Stable weight (n=23)** | **Weight loss (n=12)** | **p** |  | **Weight gain (n=8)** | **Stable weight (n=8)** | **Weight loss (n=8)** | **p** |
| Age (years) | 57.0 (8.24) | 61.6 (10.8) | 61.5 (11.3) | 0.235 |  | 58.5 (9.32) | 61.75 (7.07) | 63.3 (8.07) | 0.507 |
| **Metabolic Factors** |  |  |  |  |  |  |  |  |  |
| Weight (kg) | 84.0 (13.6) | 81.4 (17.5) | 84.1 (18.9) | 0.838 |  | 73.3 (9.54) | 72.7 (8.20) | 73.0 (20.1) | 0.996 |
| BMI (kg/m^2^) | 26.8 (3.94) | 25.9 (4.66) | 26.4 (4.98) | 0.823 |  | 26.7 (2.54) | 26.86 (3.45) | 26.7 (5.74) | 0.997 |
| Fat Mass (kg) | 29.1 (10.6) | 23.6 (12.8) | 24.4 (14.1) | 0.281 |  | 31.0 (9.54) | 32.27 (6.73) | 32.0 (11.7) | 0.963 |
| Fat Mass (%) | 34.0 (8.71) | 27.6 (10.1) | 27.9 (11.3) | 0.067 |  | 41.5 (8.58) | 44.18 (6.41) | 43.3 (5.58) | 0.737 |
| Fat Free Mass (kg) | 54.9 (8.01) | 57.8 (8.85) | 59.7 (10.9) | 0.289 |  | 42.3 (3.42) | 40.43 (5.53) | 40.8 (10.0) | 0.844 |
| Estimated Energy Intake (kcal/day) | 2472 (553) | 2629 (578) | 2715 (472) | 0.416 |  | 1859 (398) | 1754 (445) | 1880 (393) | 0.855 |
| Resting Energy Expenditure (kcal/day) | 1803 (320) | 1707 (389) | 1779 (335) | 0.638 |  | 1325 (299) | 1269 (173) | 1251 (239) | 0.834 |
| Metabolic Index (S4) | 1.10 (0.23) | 1.00 (0.17) | 1.02 (0.11) | 0.194 |  | 0.96 (0.19) | 0.93 (0.07) | 0.94 (0.16) | 0.938 |
| **Clinical Factors** |  |  |  |  |  |  |  |  |  |
| ALSFRS-R | 38.4 (4.92) | 38.4 (5.33) | 38.5 (3.80) | 0.998 |  | 37.3 (7.40) | 36.75 (4.27) | 34.8 (6.76) | 0.706 |
| Respiratory sub-score | 11.0 (1.72) | 10.7 (1.66) | 10.5 (1.68) | 0.731 |  | 11.6 (0.74) | 11.0 (1.77) | 8.63 (2.62) | 0.01 |
| Bulbar sub-score | 10.7 (2.08) | 9.26 (2.32) | 8.50 (3.75) | 0.05 |  | 10.6 (1.92) | 10.25 (1.28) | 9.13 (1.96) | 0.227 |
| Lower Limb sub-score | 4.09 (1.81) | 5.65 (2.06) | 6.00 (1.60) | 0.005 |  | 4.13 (2.17) | 4 (2.00) | 5.13 (2.30) | 0.531 |
| Upper Limb sub-score | 6.43 (2.00) | 6.52 (1.50) | 6.83 (1.59) | 0.808 |  | 6.00 (2.33) | 6.13 (2.10) | 6.00 (1.31) | 0.989 |
| Bulbar Onset (%) | 3 (13.0) | 10 (43.5) | 4 (33.3) | 0.076 |  | 2 (25.0) | 2 (25.0) | 3 (37.5) | 1 |
| ∆FRS | 0.35 (0.25-0.50) | 0.47 (0.32-0.69) | 0.68 (0.43-1.17) | 0.034 |  | 0.54 (0.37-1.06) | 0.70 (0.43-0.79) | 0.85 (0.48-1.39) | 0.672 |
| Time Since Symptom Onset (months) | 24 (15.5-34.0) | 16.0 (12.0-21.5) | 14.0 (9.0-20.3) | 0.033 |  | 20.0 (17.0-21.5) | 17.5 (15.0-20.5) | 14.5 (13.5-16.3) | 0.534 |
| Diagnostic Delay (months) | 17.0 (9.0-22.5) | 11.0 (5.50-15.5) | 7.0 (4.75-10.8) | 0.091 |  | 11.0 (9.25-15.5) | 12.0 (6.25-13.8) | 12.0 (9.75-12.5) | 0.855 |
| Riluzole Usage | 16 (69.6) | 13 (56.5) | 5 (41.7) | 0.258 |  | 3 (37.5) | 3 (37.5) | 4 (50.0) | 1.000 |
| Participants in the nested cohort were categorized by six-month weight change, adapted from GLIM weight-loss criteria, as Weight Gain (>0%), Stable Weight (0–5% loss), or Weight Loss (≥5% loss) during the first six months following study inclusion. Data are presented as mean (SD) for continuous variables, n (%) for categorical variables, and median (IQR) for time variables. P values were calculated using ANOVA with Tukey’s post-hoc tests for continuous variables, Chi-square or Fisher’s exact tests for categorical variables, and Brown-Mood median tests for time variables. ^a^Baseline metabolic data were unavailable for n=2 participants with ALS. Abbreviations: ALS, amyotrophic lateral sclerosis; ALSFRS-R, ALS Functional Rating Scale-Revised; BMI, body mass index; ΔFRS, monthly rate of loss of total ALSFRS-R points since symptom onset. | | | | | | | | | |

| **Supplementary Table 4.** Raw values used to assess concordance between equation-derived estimated energy requirements, reported estimated energy intake, and six-month weight-change category in people living with ALS (plwALS). | | | | | | | | | | | |
| --- | --- | --- | --- | --- | --- | --- | --- | --- | --- | --- | --- |
|  | **Weight Gain (n=31)** | | | **Stable Weight (n=31)** | | | **Weight Loss (n=20)** | | |  |  |
|  | **plwALS (n)** | **%EEI** | **Concordant,**  **n (%)** | **plwALS (n)** | **%EEI** | **Concordant,**  **n (%)** | **plwALS (n)** | **%EEI** | **Concordant,**  **n (%)** |  | **Overall**  **Concordance (%)** |
| **IBW 30 (30)** | 31 | 118 | 2 (6.45) | 31 | 113 | 10 (32.3) | 20 | 112 | 12 (60.0) |  | 29.3 |
| **Model 8** | 30 | 108 | 6 (20.0) | 31 | 105 | 12 (38.7) | 20 | 104 | 8 (40.0) |  | 32.1 |
| **Model 6** | 30 | 106 | 7 (23.3) | 31 | 101 | 9 (29.0) | 20 | 99.6 | 8 (40.0) |  | 29.6 |
| **Shimizu** | 31 | 106 | 4 (12.9) | 31 | 97.8 | 7 (22.6) | 20 | 95.8 | 6 (30.0) |  | 20.7 |
| **IBW 25 (30)** | 31 | 98.4 | 9 (29.0) | 31 | 94.4 | 6 (19.4) | 20 | 94.1 | 6 (30.0) |  | 25.6 |
| **MSJ 4** | 30 | 84.3 | 9 (30.0) | 31 | 85.0 | 10 (32.3) | 20 | 86.4 | 3 (15.0) |  | 27.2 |
| **IBW 22 (30)** | 31 | 86.6 | 9 (29.0) | 31 | 83.1 | 5 (16.1) | 20 | 82.8 | 3 (15.0) |  | 20.7 |
| **MSJ 5** | 30 | 88.7 | 6 (20.0) | 31 | 83.1 | 6 (19.4) | 20 | 82.3 | 2 (10.0) |  | 17.3 |
| **REE x 1.2** | 30 | 87.7 | 8 (26.7) | 31 | 79.6 | 8 (15.8) | 19 | 79.8 | 1 (5.26) |  | 21.3 |
| **REE x 1.1** | 30 | 80.4 | 11 (36.7) | 31 | 73.0 | 6 (19.4) | 19 | 73.2 | 1 (5.26) |  | 22.5 |
| **IBW 25 (25)** | 31 | 82.0 | 8 (25.8) | 31 | 78.7 | 6 (19.4) | 20 | 78.4 | 1 (5.26) |  | 18.3 |
| **Increased REE** | 31 | 79.7 | 13 (41.9) | 31 | 78.4 | 8 (25.8) | 20 | 79.1 | 0 (0.00) |  | 25.6 |
| **IBW 22 (25)** | 31 | 72.2 | 11 (35.5) | 31 | 69.2 | 4 (12.9) | 20 | 69.0 | 0 (0.00) |  | 18.3 |
